# Multiple Organ Failure as a Strong Predictor of Mortality in Patients with Hypoxic Hepatitis

**DOI:** 10.3390/jcm14155286

**Published:** 2025-07-26

**Authors:** Ji Yoon Kwak, Hankyu Jeon, Hyeon Uk Kwon, Jae Eun Kim, Seong Je Kim, Ji Hee Han, Ra Ri Cha, Jae Min Lee, Sang Soo Lee

**Affiliations:** 1 Department of Internal Medicine, Gyeongsang National University Changwon Hospital, Gyeongsang National University School of Medicine, Changwon 51472, Republic of Korea; jiuni_01@naver.com (J.Y.K.); polaris739@naver.com (H.J.); rivalharry@naver.com (H.U.K.); legenduc@naver.com (J.E.K.); hanjisky@naver.com (J.H.H.); rari83@naver.com (R.R.C.); 01179jm@naver.com (J.M.L.); 2 Institute of Health Sciences, Gyeongsang National University, Jinju 52727, Republic of Korea; 3 Department of Internal Medicine, Gyeongsang National UniversityHospital, Gyeongsang National University School of Medicine, Jinju 52727, Republic of Korea; sjhope77@naver.com

**Keywords:** hypoxic hepatitis, predisposing condition, multiple organ failure, mortality, sequential organ failure assessment

## Abstract

**Background**: Hypoxic hepatitis contributes to the development and progression of multiple organ failure (MOF). We evaluated whether MOF is associated with 30-day mortality in patients with hypoxic hepatitis. **Methods**: This retrospective study included 1011 patients diagnosed with hypoxic hepatitis at two centers in South Korea between 2010 and 2021. Organ failure was defined as a sequential organ failure assessment score ≥ 3 for each individual organ system. **Results**: Circulatory failure was the most common organ failure (n = 521), followed by respiratory (n = 380), cerebral (n = 307), renal (n = 236), coagulation (n = 182), and hepatic failure (n = 73). The proportions of patients without organ failure, with single organ failure, and with MOF were 28.7%, 22.3%, and 49.1%, respectively, with corresponding 30-day mortality rates of 17.9%, 29.3%, and 70.0%. In the multivariate Cox regression model, the presence of MOF grade 1 (two organ failures), grade 2 (three organ failures), and grade 3 (≥four organ failures) increased the risk of 30-day mortality by approximately threefold, fourfold, and fivefold, respectively, compared to patients without MOF. **Conclusions**: MOF is frequently observed in patients with hypoxic hepatitis and is a strong independent predictor of short-term mortality.

## 1. Introduction

Hypoxic hepatitis is an acute liver injury characterized by a massive but transient elevation in serum aminotransferases, accompanied by centrilobular liver cell necrosis. This form of liver injury occurs in critical conditions involving hemodynamic instability, such as circulatory shock, cardiac dysfunction, respiratory dysfunction, or sepsis [1]. The incidence of hypoxic hepatitis has been estimated at two cases per 1000 hospital admissions and two to three cases per 100 intensive care unit admissions [2,3].

In-hospital mortality in hypoxic hepatitis has been reported to exceed 50% [2,3,4,5,6]. Previous studies have shown that the extent of hepatic injury—reflected in elevated bilirubin, prothrombin time-international normalized ratio (PT-INR), and lactate dehydrogenase (LDH) levels, is associated with increased mortality [5,7]. Jager et al. reported that jaundice is also associated with increased mortality [3,6]. Other studies have identified the severity of underlying illness, the sequential organ failure assessment (SOFA) score, and the presence of multiple organ failure (MOF) as contributors to elevated mortality risk [3,8]. Therefore, both the occurrence of organ failure and the degree of hepatic injury may predict mortality in patients with hypoxic hepatitis.

A documented hypotensive event alone does not result in hypoxic hepatitis [9]. This condition develops in the presence of predisposing factors such as circulatory shock, cardiac dysfunction, respiratory dysfunction, or sepsis, and frequently coexists with failures of organ systems including hepatic, renal, cerebral, coagulation, circulatory, and respiratory systems [10]. Hypoxic hepatitis may represent a hepatic manifestation of organ failure, and the presence of organ failure is a key determinant of prognosis [11]. Accordingly, it was hypothesized that the prognosis of hypoxic hepatitis, which arises in the context of predisposing conditions, is influenced by the presence of organ failure, classified by SOFA criteria into hepatic, renal, cerebral, coagulation, circulatory, and respiratory systems. MOF, in particular, is considered a major determinant of mortality. As no universally accepted definition of organ failure in hypoxic hepatitis exists, organ failure was defined as a SOFA score ≥3 for each organ system [12,13,14]. In this study, we aimed to identify the factors associated with mortality in patients with hypoxic hepatitis and to determine whether the development of MOF influences prognosis.

## 2. Materials and Methods

### 2.1. Study Population

This was a retrospective chart review of a total of 1061 consecutive patients who met the diagnostic criteria for hypoxic hepatitis, identified from two centers between January 2010 and December 2021. The criteria were as follows: (1) predisposing conditions, including cardiac dysfunction, circulatory shock, respiratory dysfunction, sepsis, or other causes; (2) a rapid but transient elevation of aminotransferase levels exceeding 10 times the upper limit of normal (ULN) (400 U/L); and (3) exclusion of liver injury due to other causes, such as drug-induced liver injury or acute viral hepatitis. Of these, 50 patients met one or more of the following exclusion criteria: age < 18 years (n = 2), insufficient data (n = 18), or loss to follow-up (n = 30). The remaining 1011 patients were included in the final analysis.

### 2.2. Data Collection

Baseline demographic data, including age, sex, and comorbidities, were collected from electronic medical records. Clinical variables, including hepatic decompensation events (ascites, variceal hemorrhage, and hepatic encephalopathy) and infections, were reviewed. Predisposing conditions for hypoxic hepatitis, including circulatory shock, cardiac dysfunction, respiratory dysfunction, and sepsis, were assessed. Laboratory data at baseline included aspartate aminotransferase (AST), alanine aminotransferase (ALT), albumin, bilirubin, alkaline phosphatase, LDH, creatinine, PT-INR, and platelet count.

### 2.3. Definitions of Predisposing Conditions

Predisposing conditions for hypoxic hepatitis were classified as circulatory shock, cardiac dysfunction, respiratory dysfunction, and sepsis. Circulatory shock was defined as a reduction in intravascular volume due to dehydration (e.g., from burns, acute pancreatitis, vomiting, or diarrhea) or hemorrhage (e.g., from acute gastrointestinal bleeding or trauma). Cardiac dysfunction was characterized by passive congestion or reduced cardiac output resulting from acute cardiac events such as myocardial infarction, valvular heart disease, unstable arrhythmia, cardiac tamponade, pulmonary embolism, or decompensated heart failure. Respiratory dysfunction was defined as severe hypoxemia caused by pneumonia or acute exacerbation of chronic respiratory failure, including interstitial lung disease, pleural fibrosis, and chronic obstructive pulmonary disease. Sepsis was defined as a documented infection or positive blood culture in combination with at least two of the following SIRS criteria: temperature > 38 °C or <36 °C, heart rate > 90/min, respiratory rate > 20/min or PaCO_2_ < 32 mmHg, leukocyte count > 12,000/mm^3^ or <4000/mm^3^, or >10% immature neutrophils [15]. Other conditions were defined as those not falling into the aforementioned categories (e.g., sleep apnea, toxigenic etiologies, or unknown causes). For patients with multiple predisposing conditions, the condition considered to have the greatest impact on hypoxic hepatitis was determined by two experienced hepatologists. For statistical analysis, pneumonia with concurrent sepsis was categorized as respiratory dysfunction.

### 2.4. Definitions of Organ Failure

The diagnostic criteria for organ failure were defined as a SOFA score ≥ 3 for each organ system (Supplementary Table S1). Specifically, liver failure was defined as a bilirubin level ≥ 6 mg/dL; renal failure as a creatinine level ≥ 3.5 mg/dL or the need for renal replacement therapy; cerebral failure as a Glasgow Coma Scale score ≤ 9; coagulation failure as a platelet count < 50,000/mm^3^; circulatory failure as the use of vasopressors, specifically dopamine > 5 μg/kg/min or any dose of epinephrine or norepinephrine; and respiratory failure as a PaO_2_/FiO_2_ ratio <200 mmHg with respiratory support. MOF was defined as failure of two or more organ systems based on a SOFA score ≥ 3 for each involved system.

### 2.5. Statistical Analysis

Continuous variables were presented as medians (interquartile range), and categorical variables as number (%). Non-parametric tests, including the Mann–Whitney U test, were used for non-normally distributed continuous variables. The chi-squared or Fisher exact test for categorical variables. To identify predictors of 30-day mortality, univariate and multivariate analyses were conducted using the Cox proportional hazards regression model. Risk was reported as a hazard ratio (HR) with 95% confidence intervals. A two-tailed *p* value < 0.05 was considered statistically significant. Statistical analyses were conducted using IBM SPSS Statistics version 24 (IBM Corp., Armonk, NY, USA).

### 2.6. Ethics Statement

The study was conducted in accordance with the principles outlined in the 1964 Declaration of Helsinki and approved by the institutional review boards of Gyeongsang National University Changwon Hospital (IRB File No. 2021-06-032) and Gyeongsang National University Hospital (IRB File No. 2015-07-029).

## 3. Results

### 3.1. Patient Characteristics

The baseline characteristics of the 1011 patients with hypoxic hepatitis are summarized in Table 1. The median age was 69 years, and 60.4% of the patients were male. At the time of diagnosis, 12.9% had cirrhosis and 9.0% had hepatic decompensation. Predisposing conditions included circulatory shock (14.6%), cardiac dysfunction (37.1%), respiratory dysfunction (16.6%), sepsis (29.0%), and other causes (2.7%). At diagnosis, liver, renal, cerebral, coagulation, circulatory, and respiratory failure were observed in 73, 236, 307, 182, 521, and 380 patients, respectively, and 290 patients presented with no organ failure. A total of 54.9% required treatment in the intensive care unit. The median AST and ALT levels at diagnosis were 838 and 489 U/L, respectively.

### 3.2. Organ Failure and Mortality

Among the 1011 patients with hypoxic hepatitis, 290 (28.7%) had no organ failure, 225 (22.3%) had single-organ failure, and 496 (49.1%) had MOF (Table 2). Among the 225 patients with single-organ failure, circulatory failure was most common (87, 38.7%), followed by renal (37, 16.4%), respiratory (33, 14.7%), liver (18, 14.6%), coagulation (29, 12.9%), and cerebral (21, 9.3%) failure.

**Figure 1:**
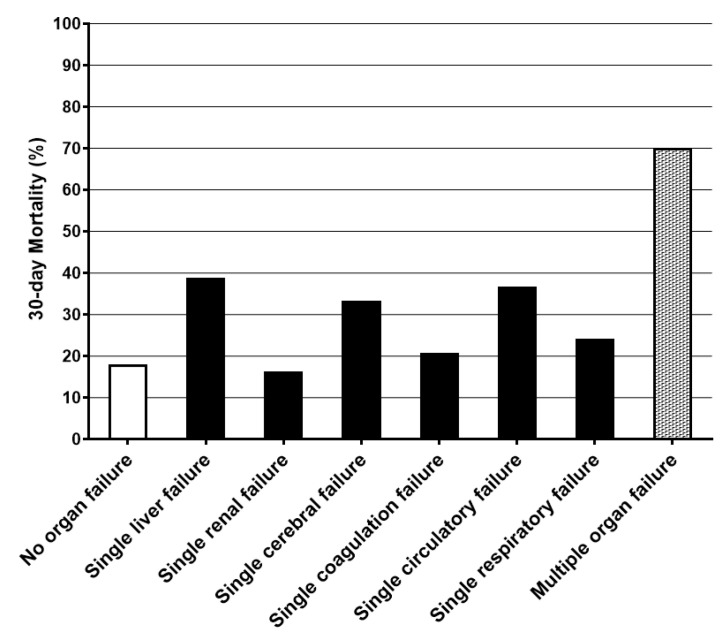
Thirty-day mortality according to the types of organ failure.

The 30-day mortality rate was 46% in the overall cohort. Patients with single-organ failure had a significantly higher 30-day mortality rate (66/225, 29.3%) than those without organ failure (52/290, 17.9%; *p* = 0.003). However, the 30-day mortality rate did not differ significantly between patients with each type of single-organ failure (excluding single circulatory failure) and those without organ failure (Figure 1, Table 2). In contrast, the 30-day mortality rate was significantly higher in patients with MOF (347/496, 70.0%) than in those with 0 or 1 organ failure (118/515, 22.9%; *p* < 0.001). These findings indicate that MOF is a key determinant of mortality in hypoxic hepatitis.

### 3.3. Multiple Organ Failure in Hypoxic Hepatitis

**Figure 2:**
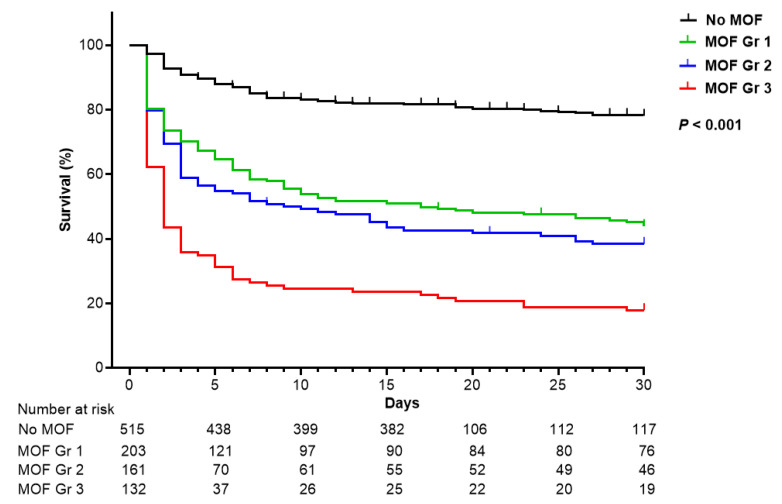
Kaplan–Meier curve for survival according to the multiple organ failure grade. *MOF grades: Grade 0 (no MOF), Grade 1 (two organ failure), Grade 2 (three organ failure), and Grade 3 (four organ failure or more).

A strong stepwise relationship was observed between the number of organ failures and the 30-day mortality rate. Based on this trend, patients were classified into four MOF grades: grade 0, no organ failure; grade 1, two organ failures; grade 2, three organ failures; and grade 3, four or more organ failures (Figure 2).

Supplementary Table S2 compares the baseline characteristics of patients with and without MOF. Despite a significantly higher mortality rate, patients with MOF were younger (68 years) than those without (70 years; *p* = 0.028). The distribution of sex, diabetes, and cirrhosis was similar in both groups. However, hepatic decompensation was more frequent in patients with MOF (12.7% vs. 5.4%; *p* < 0.001). In addition, these patients showed more severe deterioration in liver chemistry, including AST, ALT, bilirubin, LDH, albumin, and PT-INR, indicating impaired purification, synthetic function, and hepatocellular necrosis. Infection or sepsis was also more common in patients with MOF than in those without.

### 3.4. Types of Organ Failure According to Predisposing Conditions

The types of organ failure and associated mortality rates based on predisposing conditions are shown in Table 3. Among the predisposing conditions, cardiac dysfunction was associated with the lowest prevalence of MOF (36.8%) and the lowest 30-day mortality rate (38.7%). Conversely, sepsis was associated with the highest prevalence of MOF (63.8%) and the highest 30-day mortality rate (57.0%). Among the 375 patients with cardiac dysfunction, the most frequent type of single-organ failure was circulatory failure (n = 46), followed by renal (n = 26) and respiratory (n = 11) failure. These findings suggest that hypoxic hepatitis due to cardiac dysfunction is more likely to remain limited to no or single-organ failure. In contrast, hypoxic hepatitis associated with sepsis tends to progress to MOF and is linked with higher mortality.

Among these 293 patients with sepsis, 36.2% had no MOF, while 22.2%, 17.4%, and 24.2% had MOF grades 1, 2, and 3, respectively. Additionally, 63.5% received vasopressor support, 41.0% underwent mechanical ventilation, 29.0% received renal replacement therapy, and 61.8% were admitted to the intensive care unit. The causative pathogens of sepsis patients are listed in Supplementary Table S3.

### 3.5. Predictors Associated with 30-Day Mortality

A Cox proportional hazards regression model was used to assess predictors of 30-day mortality (Table 4). In this analysis, circulatory shock, cardiac dysfunction, and other conditions were used as reference categories for predisposing conditions, and grade 0 was used as the reference for MOF grades. In univariate analysis, age, respiratory dysfunction and sepsis, AST level, PT-INR, hepatic decompensation, and MOF grades 1, 2, and 3 were significant factors affecting mortality. In multivariate analysis, age (adjusted HR = 1.018), AST level (adjusted HR per 100 U/L = 1.004), albumin level (adjusted HR = 0.656), PT-INR (adjusted HR = 1.061), MOF grade 1 (adjusted HR = 2.866), grade 2 (adjusted HR = 3.912), and grade 3 (adjusted HR = 5.008) were independently associated with 30-day mortality.

## 4. Discussion

In this study, we aimed to determine whether MOF, as defined by the SOFA system, substantially influences mortality in patients with hypoxic hepatitis. In previous studies, organ dysfunction was defined as a SOFA score of 1 or 2, while organ failure was defined as a SOFA score of 3 or higher to assess prognosis in intensive care unit patients [12,14,16]. However, no universally accepted definition of organ failure exists for patients with hypoxic hepatitis. Therefore, in this study, organ failure was defined as a SOFA score ≥3 for each organ system.

The present findings share certain similarities with prior research while also offering distinctions [2,3,4,5,6,7,8,11,17]. The overall 30-day mortality rate observed was 46%, which is comparable to or slightly lower than the 45–72% range reported previously. The laboratory criteria for AST or ALT in the diagnosis of hypoxic hepatitis have been controversial. Henrion et al. proposed a cutoff of more than 20 times the ULN, while other studies have suggested cutoff values ranging from 2.5 to 10 times the ULN (Table 5). Most existing studies have focused primarily on hypoxic hepatitis within ICU settings. However, one advantage of our study is that it includes both ICU (54.9%) and non-ICU (45.1%) settings. Clinically, hypoxic hepatitis is frequently observed in non-ICU environments even when AST or ALT levels do not exceed 20 times the ULN. To enhance diagnostic sensitivity, hypoxic hepatitis was defined as aminotransferase levels exceeding 10 times the ULN. Independent predictors of 30-day mortality identified in this study—age, AST level, PT-INR, albumin level, and presence of MOF—were consistent with those reported in earlier studies. In particular, this study systematically presented mortality rates by organ failure status based on the SOFA system. Predisposing or underlying conditions associated with hypoxic hepatitis are classified using various criteria. Here, for clarity and simplification, predisposing conditions were categorized into five groups: circulatory shock, cardiac dysfunction, respiratory dysfunction, sepsis, and others.

The findings suggest that MOF is a strong prognostic indicator in hypoxic hepatitis, with an overall prevalence of 50%. Among affected patients, the prevalence rates of no organ failure, single-organ failure, and MOF were 28.7%, 22.3%, and 49.1%, respectively. Corresponding 30-day mortality rates were 17.9% for no organ failure, 29.3% for single-organ failure, and 70.0% for MOF. A key finding was the graded association between MOF severity and mortality, with MOF grades 1, 2, and 3 increasing the risk of 30-day mortality approximately threefold, fourfold, and fivefold, respectively, compared to patients without MOF. These results are consistent with previous reports identifying the SOFA score as a robust predictor of mortality in hypoxic hepatitis [5,21].

The main predisposing conditions examined in the current study, circulatory shock, cardiac dysfunction, respiratory dysfunction, and sepsis, account for over 90% of hypoxic hepatitis cases [1,10,18]. The pathophysiology of liver cell damage in hypoxic hepatitis varies according to the predisposing condition. In cardiac dysfunction, the hemodynamic mechanism involves ischemia due to passive hepatic congestion and reduced hepatic blood flow [18,22]. In circulatory shock, the hemodynamic pattern is characterized solely by liver ischemia resulting from systemic hypoperfusion [23]. In sepsis, the proposed mechanism involves impaired oxygen utilization by hepatocytes, which are unable to meet the elevated oxygen demand [24,25,26]. Inflammatory mediators and endotoxins reduce hepatocyte oxygen extraction from the blood, leading to cellular necrosis. In cardiac dysfunction, splanchnic blood flow is diminished while oxygen extraction remains intact. Conversely, in sepsis, splanchnic blood flow is elevated, but oxygen extraction by hepatocytes is impaired [18]. Liver ischemia, due to circulatory shock and cardiac dysfunction, and hypoxemia, secondary to respiratory dysfunction and sepsis, play a critical role in the development of hypoxic hepatitis [1,8,9,19]. Nevertheless, a documented shock state is observed in only approximately half of patients with hypoxic hepatitis. In the current cohort, cardiac dysfunction and circulatory shock primarily induced liver ischemia and were associated with a lower frequency of MOF. In contrast, respiratory dysfunction and sepsis primarily contributed to hypoxic liver injury and were associated with a higher incidence of MOF. These findings suggest that, based on the hemodynamic mechanisms of hypoxic hepatitis, the presence of MOF significantly affects patient prognosis.

This study had a few limitations. First, because of its retrospective design, classification of predisposing conditions and organ failure was constrained. Second, the study was conducted at only two hospitals. Nonetheless, the cohort included 1011 patients, making it one of the largest retrospective studies in this field, closely paralleling the largest recent cohort study comprising 1116 patients [4]. Additionally, because of its observational design, the study cannot determine causality between MOF and hypoxic hepatitis; therefore, MOF should be interpreted not as a cause of hypoxic hepatitis but as a useful prognostic factor for mortality in hypoxic hepatitis. Although N-acetylcysteine was not used in this cohort, it is worth noting that recent studies suggest potential benefits of N-acetylcysteine in non-acetaminophen liver injuries and hypoxic hepatitis [27,28].

## 5. Conclusions

These findings highlight the prognostic value of MOF, assessed using the SOFA system, in hypoxic hepatitis. Future prospective studies are warranted to confirm whether the presence of MOF increases mortality in this patient population.

## Figures and Tables

**Table 1:** Baseline characteristics of patients with hypoxic hepatitis (N = 1011).

Characteristics	N = 1011
Age, years	69.0 (56.0–78.0)
Male gender	611 (60.4%)
Diabetes	286 (28.3%)
Liver cirrhosis	130 (12.9%)
Hepatic decompensation	91 (9.0%)
Infection	295 (29.2%)
Vasopressor support	550 (54.4%)
Mechanical ventilation	380 (37.6%)
Renal replacement therapy	243 (24.0%)
Admission at the intensive care unit	555 (54.9%)
Predisposing conditions	
Circulatory shock	148 (14.6%)
Cardiac dysfunction	375 (37.1%)
Respiratory dysfunction	168 (16.6%)
Sepsis	293 (29.0%)
Others	27 (2.7%)
Type of organ failures	
Liver failure	73 (7.2%)
Renal failure	236 (23.3%)
Cerebral failure	307 (30.4%)
Coagulation failure	182 (18.0%)
Circulatory failure	521 (51.5%)
Respiratory failure	380 (37.6%)
No organ failure	290 (28.7%)
Initial laboratory values	
AST, U/L	838.0 (557.0–1720.0)
ALT, U/L	489.0 (251.0–882.0)
Albumin, g/dL	2.8 (2.3–3.3)
Bilirubin, mg/dL	1.4 (0.8–2.6)
ALP, U/L	102.0 (71.0–161.0)
LDH, U/L *	1042.0 (557.0–2046.0)
Creatinine, mg/dL	1.48 (0.97–2.30)
PT-INR	1.57 (1.24–2.14)
Platelet, ×10^9^/L	130.0 (66.0–204.0)
Median AST/ALT ratio	2.0 (1.3–3.5)
Median LDH/AST ratio	1.1 (0.7–1.9)
Median LDH/ALT ratio	2.3 (1.0–4.7)
Peak laboratory values	
AST, U/L	1316.0 (664.0–2699.0)
ALT, U/L	664.0 (344.0–1439.0)
Median AST/ALT ratio	2.0 (1.3–3.6)

AST, aspartate aminotransferase; ALT, alanine aminotransferase; ALP, Alkaline Phosphatase; LDH, lactate dehydrogenase; PT-INR, prothrombin time- international normalized ratio. * Data are missing for some patients. Data are presented as the medians (interquartile range) for continuous data and percentages for categorical data.

**Table 2:** Prevalence and 30-day mortality of organ failures in patients with hypoxic hepatitis (N = 1011).

Number of Organ Failures	Prevalence (%)	30-Day Mortality (%)	*p*
No organ failure	290 (28.7%)	52 (17.9%)	Reference
Single organ failure	225 (22.3%)	66 (29.3%)	0.003
Liver failure	18	7 (38.9%)	0.056
Renal failure	37	6 (16.2%)	1.000
Cerebral failure	21	7 (33.3%)	0.089
Coagulation failure	29	6 (20.7%)	0.800
Circulatory failure	87	32 (36.8%)	<0.001
Respiratory failure	33	8 (24.2%)	0.353
Multiple organ failure	496 (49.1%)	347 (70.0%)	<0.001
Two organ failures	203	122 (60.1%)	<0.001
Three organ failures	161	112 (69.6%)	<0.001
Four organ failures or more	132	113 (85.6%)	<0.001

The *p* value refers to the values obtained by comparing each type of organ failure patient to those with no organ failure as the reference group.

**Table 3:** Types of organ failure and mortality according to predisposing condition of hypoxic hepatitis (N = 1011).

Predisposing Condition	No Organ Failure	Single Organ Failure	Multiple Organ Failure	30-Day Mortality
Circulatory shock (n = 148)	44 (29.7%)	28 (18.9%)	76 (51.3%)	58 (39.2%)
Cardiac dysfunction (n = 375)	140 (37.3%)	97 (25.9%)	138 (36.8%)	145 (38.7%)
Respiratory dysfunction (n = 168)	38 (22.6%)	41 (24.4%)	89 (53.0%)	90 (53.6%)
Sepsis (n = 293)	51 (17.4%)	55 (18.8%)	187 (63.8%)	167 (57.0%)
Others (n = 27)	17 (63.0%)	4 (14.8%)	6 (22.2%)	5 (18.5%)

**Table 4:** Predictors of 30-day mortality of patients with hypoxic hepatitis.

Variable	Univariate Analysis		Multivariate Analysis	
	*p*	HR (95% CI)	*p*	HR (95% CI)
Age, per year	<0.001	1.012 (1.005–1.018)	<0.001	1.018 (1.011–1.025)
Predisposing conditions				
Circulatory shock/cardiac dysfunction/others	reference		reference	
Respiratory dysfunction/sepsis	<0.001	1.604 (1.335–1.926)	0.586	1.056 (0.869–1.283)
AST per 100 U/L	<0.001	1.009 (1.006–1.013)	0.040	1.004 (1.000–1.007)
Albumin per g/dL	<0.001	0.447 (0.389–0.514)	<0.001	0.656 (0.562–0.766)
PT-INR	<0.001	1.098 (1.056–1.142)	0.020	1.061 (1.009–1.114)
Hepatic decompensation	<0.001	1.736 (1.331–2.265)	0.214	1.194 (0.903–1.579)
MOF grades *				
Grade 0	reference		reference	
Grade 1	<0.001	3.397 (2.636–4.378)	<0.001	2.866 (2.206–3.726)
Grade 2	<0.001	4.619 (3.562–5.990)	<0.001	3.912 (2.975–5.145)
Grade 3	<0.001	6.936 (5.335–9.017)	<0.001	5.008 (3.706–6.768)

Abbreviation: HR, hazard ratio; CI, confidence interval; AST, aspartate aminotransferase; PT-INR, prothrombin time- international normalized ratio; MOF, multiple organ failure. * MOF grades: Grade 0 (no MOF), Grade 1 (two organ failure), Grade 2 (three organ failure), and Grade 3 (four organ failure or more).

**Table 5:** Summary of Hypoxic Hepatitis Studies Incorporating Various AST or ALT Cutoff Values.

Author	Year	N	Population	AST or ALT Cutoff (U/L)	Mortality
Henrion [18]	2003	142	ICU	>800	53%
Birrer [19]	2007	322	ICU	>400	45%
Tapper [2]	2015	1782	Meta-analysis	>300	50%
Aboelsoud [3]	2017	565	ICU	>800	44%
Broecke [4]	2018	1116	ICU	>155 (females) >185 (males)	45%
Allam [20]	2024	108	Cirrhosis	>1000	73%

Abbreviation: ICU; intensive care unit.

## Data Availability

The datasets generated and/or analyzed during the current study are not publicly available due to ethical and confidentiality reasons but are available from the corresponding author on reasonable request under the Gyeongsang National University Changwon Hospital and Gyeongsang National University Hospital Ethics Committee’s approval. The data that support the findings of this study are available on request to the correspondence author. (Sang Soo Lee, Email: 3939lee@naver.com).

## Supplementary Materials

The following supporting information can be downloaded at: https://www.mdpi.com/article/10.3390/jcm14155286/s1, Table S1: The original sequential organ failure assessment score; Table S2: Baseline characteristics according to the presence of multiple organ failure. Table S3. Causative pathogens of sepsis (n = 293).
