# Supplementary material for: Multiple Organ Failure as a Strong Predictor of Mortality in Patients with Hypoxic Hepatitis
Source: J Clin Med. 2025 Jul 26;14(15):5286. doi: 10.3390/jcm14155286 (PMC12347218; doi:10.3390/jcm14155286)
Supplement: Supplementary file 1 [file jcm-14-05286-s001.zip › jcm-3731629-supplementary.pdf]

Supp. Table 1. The original sequential organ failure assessment score

Organ/System	Score	0	1	2	3	4
Liver	Bilirubin, mg/dL	<1.2	1.2–1.9	2.0–5.9	6.0–11.9	≥12.0
Renal	Creatinine, mg/dL	<1.2	1.2–1.9	2.0–3.4	3.5–4.9	≥5.0 or use of RRT
Cerebral nervous system	Glasgow coma scale	15	13–14	10–12	6–9	<6
Coagulation	Platelet × $10^3/mm^3$	≥150	<150	<100	<50	<20
Cardiovascular system	MAP or administration of vasopressors required	MAP ≥70 mmHg	MAP < 70 mmHg	Dopamine ≤ 5 μg/kg/min or dobutamine (any dose)	Dopamine > 5 or E ≤ 0.1 or NE ≤ 0.1 μg/kg/min	Dopamine > 15 or E > 0.1 or NE > 0.1 μg/kg/min
Respiration	PaO2/FiO2, mmHg	≥400	<400	<300	<200 with respiratory support	<100 with respiratory support

MAP, Mean arterial pressure; RRT, renal replacement therapy; E, epinephrine; NE, norepinephrine; PaO2, partial pressure of arterial oxygen; FiO2, fraction of inspired oxygen.

Organ failure is defined as a SOFA score of 3 or higher for individual organ system.

Supp. Table 2. Baseline characteristics according to the presence of multiple organ failure

Characteristics	No MOF	MOF	*P*
No.	515	496	
Age, year	70.0 (58.0 - 79.0)	68.0 (55.0 - 77.0)	0.028
Male gender	303 (58.8%)	308 (62.1%)	0.304
Diabetes	144 (28.0%)	142 (28.6%)	0.834
Liver cirrhosis	60 (11.7%)	70 (14.1%)	0.260
Hepatic decompensation	28 (5.4%)	63 (12.7%)	<0.001
Infection	121 (23.5%)	174 (35.1%)	<0.001
Predisposing conditions			<0.001
Circulatory shock	72 (14.0%)	76 (15.3%)	
Cardiac dysfunction	237 (46.0%)	138 (27.8%)	
Respiratory dysfunction	79 (15.3%)	89 (17.9%)	
Sepsis	106 (20.6%)	187 (37.7%)	
Others	21 (4.1%)	6 (1.2%)	
Initial laboratory values			

AST, *U/L*	759.0 (534.0 - 1490.0)	943.0 (583.0 - 2122.5)	<0.001
ALT, *U/L*	531.0 (288.0 - 917.0)	445.5 (223.0 – 844.0)	0.012
Albumin, *g/dL*	3.0 (2.6 – 3.5)	2.5 (2.1 – 3.0)	<0.001
Bilirubin, *mg/dL*	1.3 (0.8 – 2.1)	1.6 (0.8 – 3.3)	0.001
LDH, *U/L**	787.5 (455.0 – 1608.0)	1260.0 (670.0 – 2565.0)	<0.001
Creatinine, *mg/dL*	1.17 (0.77 – 1.75)	1.82 (1.26 – 2.75)	<0.001
PT-INR	1.42 (1.16 – 1.85)	1.83 (1.40 – 2.58)	<0.001
Platelet, $\times 10^9/L$	156.0 (106.0 – 225.0)	85.0 (42.0 – 162.8)	<0.001
Peak laboratory values			
AST, U/L	1017.0 (570.0 - 2299.0)	1909.0 (731.8 – 4001.5)	<0.001
ALT, U/L	647.0 (355.0 - 1226.0)	710.5 (336.3 – 1756.0)	0.047
SOFA score	4.0 (2.0 – 6.0)	13.0 (10.3 – 16.0)	<0.001

Abbreviation: MOF, multiple organ failure; ESRD, end-stage renal disease; ICU, intensive care unit; AST, aspartate aminotransferase; ALT, alanine aminotransferase; LDH, lactate dehydrogenase; PT-INR, prothrombin time- international normalized ratio; SOFA, Sequential Organ Failure Assessment.

*Data are missing for some patients.

Data are presented as the medians (interquartile range) for continuous data and percentages for categorical data.

Supp. Table 3. Causative pathogens of sepsis (n = 293)

Pathogen	N (%)
Escherichia	48 (16.4%)
Klebsiella	44 (15.0%)
Staphylococcus	33 (11.3%)
Enterococcus	22 (7.5%)
Acinetobacter	19 (6.5%)
Candida	14 (4.8%)
Pseudomonas	13 (4.4%)
Enterobacter	10 (3.4%)
Streptococcus	9 (3.1%)
Tsutsugamushi	8 (2.7%)
Clostridium	6 (2.0%)
Stenotrophomonas	5 (1.7%)
Mycobacterium tuberculosis	4 (1.4%)

Bacillus	3 (1.0%)
Corynebacterium	3 (1.0%)
Burkholderia	3 (1.0%)
Others	22 (7.5%)
No growth	59 (20.1%)
